# The effect of light conditions on interpreting oil composition engineering in Arabidopsis seeds

**DOI:** 10.1002/pld3.67

**Published:** 2018-06-26

**Authors:** Nischal Karki, Philip D. Bates

**Affiliations:** ^1^ Department of Chemistry and Biochemistry The University of Southern Mississippi Hattiesburg Mississippi

**Keywords:** hydroxylated fatty acids, light, ricinoleate, triacylglycerol

## Abstract

*Arabidopsis thaliana* is the most developed and utilized model plant. In particular, it is an excellent model for proof‐of‐concept seed oil engineering studies because it accumulates approximately 37% seed oil by weight, and it is closely related to important Brassicaceae oilseed crops. Arabidopsis can be grown under a wide variety of conditions including continuous light; however, the amount of light is strongly correlated with total seed oil accumulation. In addition, many attempts to engineer novel seed oil fatty acid compositions in Arabidopsis have reported significant reductions in oil accumulation; however, the relative reduction from the nontransgenic controls varies greatly within the literature. A set of experiments were conducted to systematically analyze the effect of light conditions (including day/night cycle vs. continuous light, and different light intensities) on the relative accumulation of seed oil between three different transgenic lines producing novel hydroxy fatty acids and their nontransgenic background. Oil content was measured per seed and as a percentage of seed weight. Our results indicate the relative amount of seed oil between transgenic lines and nontransgenic controls is dependent on both the light conditions and the type of oil content measurement utilized. In addition, the light conditions effect the relative accumulation of the novel fatty acids between various transgenic lines. Therefore, the success of novel fatty acid proof‐of‐concept engineering strategies on both oil accumulation and fatty acid composition in Arabidopsis seeds should be considered in light of the select growth and measurement conditions prior to moving engineering strategies into crop plants.

## 1 INTRODUCTION

The fatty acids within triacylglycerols (TAGs, oils) are the most energy dense form of biological carbon storage. TAGs which accumulate in the seeds of oilseed crops are an important nutritional source for both calories and essential fatty acids required in human diets. In addition, seed oils also represent a renewable resource for industrial chemicals and biofuels (Carlsson, Yilmaz, Green, Stymne, & Hofvander, 2011; Durrett, Benning, & Ohlrogge, 2008; Dyer, Stymne, Green, & Carlsson, 2008). However, many of the most nutritionally valuable or industrially useful fatty acids do not accumulate in major oilseed crops, but accumulate in microalgae or in terrestrial plants with poor agronomic features (Badami & Patil, 1980; Gunstone, Harwood, & Dijkstra, 2007). Therefore, the bioengineering of oilseed crops to accumulate TAG with novel fatty acid compositions for use in food or industrial feedstocks has been a goal of the plant lipid community for over 20 years. Most engineering of plants to produce novel TAG fatty acid compositions has been first demonstrated in *Arabidopsis thaliana* seeds, and the wide range of unique fatty acid compositions produced has been reviewed extensively (Aznar‐Moreno & Durrett, 2017; Bates, 2016; Cahoon et al., 2007; Carlsson et al., 2011; Dyer et al., 2008; Haslam et al., 2013; Lee, Chen, & Kim, 2015; Lu, Napier, Clemente, & Cahoon, 2011; Napier, 2007; Napier, Haslam, Beaudoin, & Cahoon, 2014; Ruiz‐Lopez, Usher, Sayanova, Napier, & Haslam, 2015; Singh, Zhou, Liu, Stymne, & Green, 2005; Vanhercke, Wood, Stymne, Singh, & Green, 2013). Despite the over two decades of plant lipid engineering, we still cannot predict the effect of most engineering approaches on the final fatty acid composition or total oil amount, thereby implying that more basic research is needed to understand the factors which control both wild type and transgenic seed oil content.

As the most developed model plant species (Koornneef & Meinke, 2010; Provart et al., 2016), *Arabidopsis thaliana* is typically the first choice for most proof‐of‐concept plant metabolic engineering studies. Arabidopsis is particularly useful for engineering studies on plant seed oils because it is an oilseed plant that accumulates approximately 37% of seed weight as triacylglycerol (Li, Beisson, Pollard, & Ohlrogge, 2006). In addition, as a member of the Brassicaceae family, it is closely related to the important oilseed crop *Brassica napus* (canola/rapeseed) and the emerging biotech crop *Camelina sativa* (Iskandarov, Kim, & Cahoon, 2014). Therefore, proof‐of‐concept seed oil engineering studies in Arabidopsis could lead to relatively easy transition to actual crops once the conditions are mostly optimized in Arabidopsis.

The production of hydroxylated fatty acids (HFA) normally found in high abundance in seeds of castor (*Ricinus communis*) or several species from the genera Physaria (https://plantfadb.bch.msu.edu/) has been used many times over the past 20 years as a model for the engineering of novel fatty acids into the seed oil of Arabidopsis (reviewed in detail in: (Vanhercke et al., 2013; Lee et al., 2015; Bates, 2016; Aznar‐Moreno & Durrett, 2017)). The proportion of HFA in Arabidopsis seed oil have ranged from less than 10% (Broun, Boddupalli, & Somerville, 1998; Moire, Rezzonico, Goepfert, & Poirier, 2004) to approximately 30% (van Erp, Shockey, Zhang, Adhikari, & Browse, 2015), with most results between approximately 15% and 25% (Bates et al., 2014; Broun & Somerville, 1997; Burgal et al., 2008; Dauk, Lam, Kunst, & Smith, 2007; Lu, Fulda, Wallis, & Browse, 2006; Lunn, Wallis, & Browse, 2018; van Erp, Bates, Burgal, Shockey, & Browse, 2011). However, all engineering attempts are still far below the 90% HFA found in castor oil (Gunstone et al., 2007). Much of the variation in engineering HFA into Arabidopsis seed oil comes from the use of: different promoters to express transgenes (Broun et al., 1998; Lu et al., 2006); different fatty acid hydroxylases (e.g., *RcFAH12* or *LfFAH12* (Broun et al., 1998)); different mutant backgrounds for transformation to reduce competition for acyl substrates by endogenous enzymes (Broun et al., 1998; Dauk et al., 2007; van Erp et al., 2015); and whether or not HFA selective TAG synthesis enzymes (acyl‐CoA:diacylglycerol acyltransferase (DGAT) or phospholipid:diacylglycerol acyltransferase (PDAT)) were also coexpressed with the hydroxylase to selectively accumulate HFA in seed oil (Bates et al., 2014; Burgal et al., 2008; van Erp et al., 2011, 2015).

In addition to the change in TAG fatty acid composition, the transgenic production of HFA has been reported to reduce total seed oil accumulation by up to 50% (Bates & Browse, 2011; Bates et al., 2014; Dauk et al., 2007; Lunn et al., 2018; van Erp et al., 2011). Comparative transcriptomics and in vivo labeling assays of fatty acid synthesis have demonstrated that the reduced oil in HFA accumulating seeds is due to a biochemical reduction in acetyl‐CoA carboxylase activity, which is the first committed step of fatty acid synthesis (Bates et al., 2014). The seed oil content of HFA producing lines can be at least partially recovered by upregulating acetyl‐CoA carboxylase (Adhikari, Bates, & Browse, 2016) or by coexpression of the hydroxylase with HFA selective TAG synthesis enzymes (*RcDGAT2* or *RcPDAT1* (van Erp et al., 2011; Bates et al., 2014; van Erp et al., 2015)). The more efficient incorporation of HFA into TAG by HFA selective DGAT or PDAT apparently reduces the negative effect of HFA on lipid metabolism indicated by a recovered in vivo acetyl‐CoA carboxylase activity and more seed oil (Bates et al., 2014). However, the mechanisms that biochemically inhibit plastid localized acetyl‐CoA carboxylase activity due to the production of HFA in the endoplasmic reticulum are still unknown. Similar reduced oil content within transgenic seeds accumulating novel fatty acids has also been reported for a range of different transgenic plants and novel fatty acids produced (Knutzon et al., 1999; Larson, Edgell, Byrne, Dehesh, & Graham, 2002; Li et al., 2012; Mansour et al., 2014; Shrestha, Callahan, Singh, Petrie, & Zhou, 2016). Therefore, successful TAG composition engineering will also need to successfully recover any reduced oil penalty that novel fatty acid production has on seed oil accumulation.

While different engineering strategies obviously effect the final oil fatty acid composition and total amount, it is not clear if some the variability in HFA engineering reported in the literature may also be related to differences in growth conditions between laboratories. For example, variations in HFA content and oil quantity in the same genetic lines have been reported between different sets of experiments performed over a 10‐year span (Adhikari et al., 2016; Bates et al., 2014; Bayon, Chen, Weselake, & Browse, 2015; Burgal et al., 2008; Lu et al., 2006; Lunn et al., 2018; van Erp et al., 2011, 2015). Arabidopsis can be grown to maturity in a variety of ways (Rivero et al., 2014), in a glasshouse (with variable light/temperature) or in a growth chamber with defined conditions. Typical growth conditions are as follows: temperatures (~16–25°C), day photoperiods (~12–16 hr), light intensities ~100–400 μmol photons m^−2^ s^−1^, or even at 24‐hr light to speed up the maturation process. The effects of various conditions on wild‐type Arabidopsis growth have been well documented in: 101 ways to grow Arabidopsis https://ag.purdue.edu/hla/Hort/greenhouse/pages/101-ways-to-grow-arabidopsis.aspx. In particular for plant lipid metabolism, light is well known to affect fatty acid synthesis through thioredoxin‐linked reductive activation of plastidic acetyl‐CoA carboxylase (Browse, Roughan, & Slack, 1981; Kozaki & Sasaki, 1999; Sasaki, Kozaki, & Hatano, 1997), which effects both lipid compositions (Maatta et al., 2012) and seed oil production in developing green oilseeds (Goffman, Alonso, Schwender, Shachar‐Hill, & Ohlrogge, 2005; Li et al., 2006). In wild‐type Arabidopsis, the amount of light is one of the major factors that lead to the variation of seed oil content reported in the literature of ~24%–43% seed oil by weight and ~3–9 μg oil per seed (Li et al., 2006). The amount of light used to grow HFA accumulating transgenic Arabidopsis plants reported in the literature also varies widely (if even reported at all). A few examples are as follows: just the photoperiod length reported such as continuous light (Dauk et al., 2007), or glasshouse supplemented with lamps to 16 hr day (van Erp et al., 2011); or both photoperiod and amount of light with a range of quantities such as, 16 hr and 150 μmol photons m^−2^ s^−1^ (Lu et al., 2006), or 24 hr and ~100–200 μmole photons m^−2^ s^−1^ (van Erp et al., 2011).

Considering the effect that both novel fatty acid production and light have on seed oil synthesis, we questioned if part of the variation in the reported successes of HFA engineering could be due to differences in light conditions between experiments and between laboratories. To move a proof‐of‐concept engineering strategy tested in Arabidopsis to an actual field crop will require that the engineering strategy be robust at any growth condition and not be dependent on select laboratory conditions such as very low light or 24‐hr light conditions. Therefore, we investigated the effect of 16 versus 24‐hr photoperiods, and three different 16‐hr photoperiod light intensities on the seed oil content of three different transgenic Arabidopsis lines accumulating HFA, and the nontransgenic background line. Our results demonstrate that the interpretation of the success of multigene stacking for “increase in HFA content” and “recovery of reduced oil content” are both dependent on light conditions and the type of oil content measurement utilized to draw conclusions. These results will be valuable for designing experiments to evaluate future oilseed engineering strategies in Arabidopsis prior to oilseed crop engineering.

## 2 METHODS

### 2.1 Plant germination

The seeds were sterilized using an aqueous solution of 10% bleach, 27% ethanol, and 0.1% SDS, followed by five washes of water and final suspension in 0.1% Agar solution, and applied to germination plates (1x MS salts, 0.05% MES free acid, 1% sucrose, and 0.8% Agar, pH 5.7). The plates were incubated at 4°C for 3 days then placed under the low light condition (see below) until all lines germinated and produced two true leaves (approximately 7–10 days). The seedlings were then transferred to soil and placed in the proper light conditions for each light treatment below.

### 2.2 Growth conditions for light treatments:

All plants were grown in Percival growth chambers under white fluorescent light; the chambers did not have humidity control. The 16‐hr versus 24‐hr photoperiod experiment was grown in a model AR‐22L chamber which contains two independent growth compartments each with an interior volume of 15.7 ft^3^. Each compartment was set to 200 μmol photons m^−2^ s^−1^ light with 23/20°C for the 16/8‐hr day/night cycle and a constant 23°C for the continuous light treatment. The high and low light intensity experiment plants were grown in equivalent E41HO chambers each with an interior volume of 37.6 ft^3^. The light intensities were 112 and 364** **μmol photons m^−2^ s^−1^ in the low and high light treatments, respectively. Each chamber had the same day/night cycle of 16/8 hr and 23/20°C. All light measurements were made with a LI‐COR^®^ LI‐250A light meter with quantum sensor LI‐190 in the middle of the chamber at pot level prior to adding the plants to the chamber. In each growth chamber, the light intensity 10 inches above the pot height (average plant height) was approximately 50–60 μmol photons m^−2^ s^−1^ higher than at pot level. For each light treatment, 10–17 individual plants of each plant line were grown together randomized across the growth chamber to minimize the effects of position within the chamber. All plants were watered three times a week and fertilized once a week with Peter's NPK 20‐20‐20 fertilizer (0.957 g/l).

### 2.3 Determination of total seed oil content

Total seed lipid content was determined by direct conversion to fatty acid methyl esters and quantification by gas chromatography with flame ionization detection (GC‐FID) (Li et al., 2006). In brief, dry seeds were weighed (2–3 mg) or counted (*n* = 30) to determine the amount of FAME by dry weight or per seed. FAMEs were produced in 1 ml 5% sulfuric acid in methanol at 85°C for 1.5 hr together with 40 or 20 μg 17:0 TAG in 0.2 ml toluene as an internal standard. FAMEs were extracted by adding 0.2–0.5 ml hexane and 1.5 ml 0.88% potassium chloride. The hexane phase was analyzed by GC‐FID separated on a RESTEK Rtx^®^‐65 column (30 m, 0.25 mm ID, *df* = 0.25 μm). GC method parameters were as follows: carrier gas, He at constant linear velocity 30cm/s; temperature profile, 190°C for 2 min, increased 10°C/min to 270°C and held for 2 min.

### 2.4 Statistical analysis

Calculations for oil quantity from GC data were made using Microsoft Excel and exported to GraphPad Prism 6.01 for statistical analysis and graphical representation. For each experiment, 10–17 individual plants per plant line represent the biological replicates and the data are presented as the average and *SEM*. For statistical analysis, ordinary two‐way ANOVA test was conducted using uncorrected Fisher's LSD at 95% confidence interval. The propagation of error through ratio calculations *A*Δ*a/B*Δ*b = C*Δ*c* was done by the two formulas, where the ratio is *C = A/B,* and the standard deviation is $\Delta c=C\times \sqrt{\left({\left(a÷A\right)}^{2}+{\left(b÷B\right)}^{2}\right)}$. Statistical relevance is represented directly onto the graph using different letters to denote significant differences. Upper‐case letters are used to compare the same lines between different growth conditions while lower‐case letters are used to compare differences between plant lines within an individual growth condition. If letters are different between two bars, then there is significant difference (*p*‐value <0.05) between the two bars.

## 3 RESULTS

### 3.1 Effect of photoperiod length on novel fatty acid engineering of seed oil

To determine the effect of light conditions on both the oil amount and oil fatty acid composition of transgenic seeds producing novel fatty acids, we analyzed the seed lipid content in four different previously produced Arabidopsis lines (Table 1) grown under various light regimens. The novel fatty acids are hydroxylated fatty acids produced through the seed‐specific expression of the castor fatty acid hydroxylase (*RcFAH12*). RcFAH12 hydroxylates oleic acid (18:1) to ricinoleic acid (18:1‐OH) (Moreau & Stumpf, 1981; Vandeloo, Broun, Turner, & Somerville, 1995). In this study, all transgenic lines are in the *fae1* mutant background (Kunst, Taylor, & Underhill, 1992) which eliminates elongation of oleic acid to eicosenoic acid (20:1), accumulating approximately double the amount of 18:1 than in wild‐type Col‐0 seeds. Therefore, due to the higher amount of the 18:1 substrate that could be used by RcFAH12 to produce HFA, the *fae1* line was originally chosen as a background for HFA production in Arabidopsis seeds as a model for novel fatty acid engineering in transgenic plants (Lu et al., 2006). Therefore, in this study, the *fae1* line is our nontransgenic control. Previously, the amount of seed oil in Col‐0 and *fae1* was demonstrated to be similar (Bates et al., 2014; Kunst et al., 1992). Line CL37 expresses the *RcFAH12* alone in *fae1* (Lu et al., 2006). The DGAT line coexpresses *RcDGAT2* with *RcFAH12* in *fae1* (Burgal et al., 2008), and the PDAT line coexpresses *RcPDAT1a* with *RcFAH12* in *fae1* (van Erp et al., 2011). The lines used in this study are summarized in Table 1. Previously, the CL37 line was demonstrated to have up to a 50% reduction in total seed oil, which was partially recovered by the coexpression of *RcDGAT2* or *RcPDAT1a,* as determined from 24‐hr fluorescent light at ~100–170 μmol photons m^−2^ s^−1^ (Bates et al., 2014).

**Table 1:** Summary of Arabidopsis genotypes utilized

Name	Genotype	Reference
*fae1*	*fae1*	Kunst et al. (1992)
CL37	*fae1/RcFAH12*	Lu et al. (2006)
DGAT	*fae1/RcFAH12/RcDGAT2*	Burgal et al. (2008)
PDAT	*fae1/RcFAH12/RcPDAT1a*	van Erp et al. (2011)

**Figure 1:**
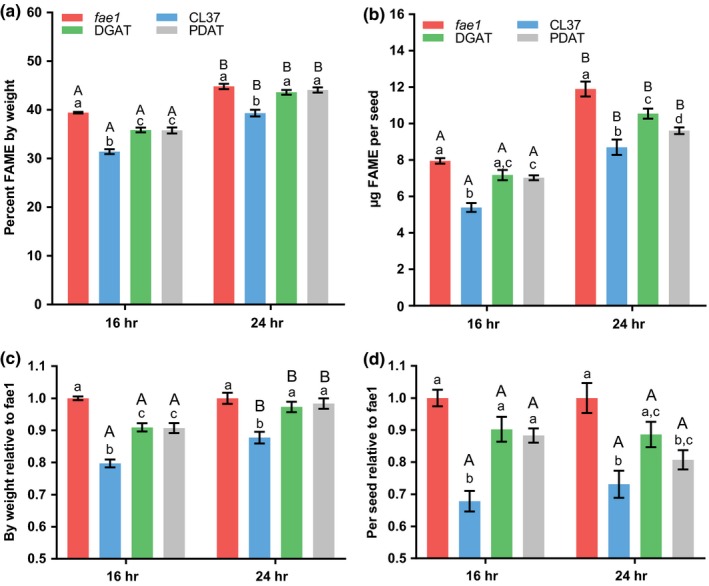
Seed oil content of day/night cycle versus continuous light. Mature seed oil content from plants grown under a 16‐hr photoperiod and 8‐dark day/night cycle versus continuous 24‐hr photoperiod. Light intensity of 200 μmol photons m^−2^ s^−1^ for both light treatments. (a) Total seed lipid content as a percentage of seed dry weight. (b) Total lipid content per seed. Data represent average and *SEM* of 10–17 biological replicates for each measurement. (c) and (d) represent the relative lipid content of the transgenic with respect to *fae1* in a and b, respectively. If letters above two bars are different, then there is significant difference between the two bars. Upper‐case letters compare the same line between light treatments; lower‐case letters compare each line within a light treatment

We initially set out to determine how growth of Arabidopsis under 24‐hr continuous light may affect our interpretation of both percent HFA within the seed oil and the recovery of the reduced oil phenotype of CL37 by coexpression of *RcDGAT2* or *RcPDAT1a*, in the DGAT and PDAT lines, respectively. Therefore, we grew all four plant lines together in two separate equivalent Percival AR22L growth chambers both set for 200 μmol photons m^−2^ s^−1^ fluorescent white light at pot height in the center of the chamber. One chamber was set to 16‐hr photoperiod (8‐hr dark), and the other to 24 hr of continuous light. 10–17 individual plants from each plant line were randomized across the growth chamber to minimize the effects of position within the chamber. The oil content of mature seeds was determined both as a percentage of seed weight (Figure 1a) and as μg per seed (Figure 1b). In general, the continuous light treatment produced more oil by both measurements (Figure 1a,b). However, the relative amount of oil in each transgenic line compared to the nontransgenic control was not consistent between light treatments or between measurement types (Figure 1c,d). When grown under the 16/8‐hr day/night cycle and measured as a percentage of seed weight, the oil content of CL37 was 80% of *fae1* and the DGAT and PDAT lines were partially recovered to 90% of *fae1* (Figure 1a,c). However, under continuous light, the CL37 oil content by weight was 90% of *fae1*, and the DGAT and PDAT lines were essentially the same as *fae1* (Figure 1a,c). Based on these results, the effect of HFA production on lowering seed oil content appears to minimal when Arabidopsis is grown at 200 μmol photons m^−2^ s^−1^ under 24‐hr light and measured as a percent of seed weight.

When the oil content was measured per seed, the differences between each transgenic and the wild‐type control were significantly greater than when measured as a percent of seed weight. CL37 oil content per seed was 68% of *fae1* for 16‐hr photoperiod samples, which was recovered to approximately 88%–90% in the DGAT and PDAT lines. For the 24‐hr light samples, CL37 was 73% of *fae1*, which was only partially recovered to 89 and 81% in the DGAT and PDAT lines, respectively (Figure 1b,d). Therefore, the 16‐hr light growth conditions and measurements of oil content per seed demonstrate both the biggest reduction in oil content caused by expression of the hydroxylase in CL37 compared to wild type, and the largest recovery in oil content (increase from 68% to 90% of wild type) when the hydroxylase is coexpressed with HFA selective acyltransferases from castor.

**Figure 2:**
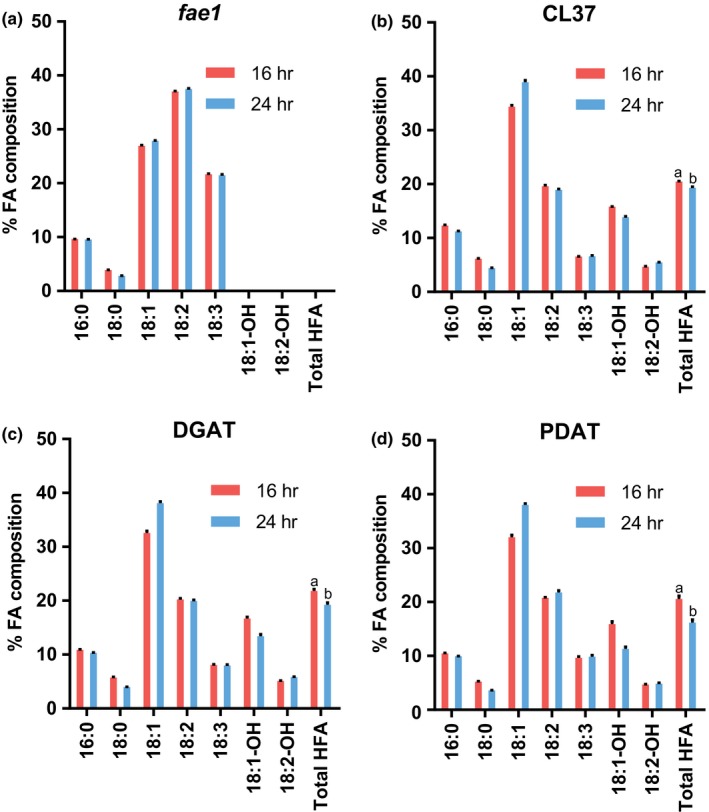
Seed fatty acid composition for day/night cycle versus continuous light. Fatty acid composition of mature seeds is percent by weight. (a) *fae1*. (b) CL37. (c) DGAT line. (d) PDAT line. Data represent average and *SEM* of 10–17 biological replicates for each measurement. The significance for total HFA content is represented by different letters above each bar. Fatty acid nomenclature: # carbons:# double bonds, ‐OH indicates presence of a hydroxyl

**Figure 3:**
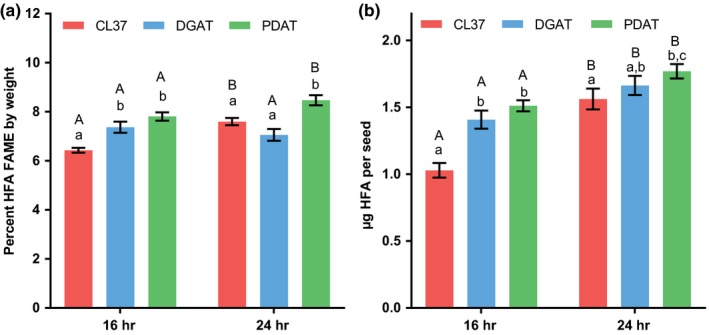
Total seed HFA accumulation for day/night cycle versus continuous light. Mature seed HFA content from plants grown under a 16‐hr photoperiod and 8‐dark day/night cycle versus continuous 24‐hr photoperiod. Light intensity of 200 μmol photons m^−2^ s^−1^ for both light treatments. (a) Total HFA content as a percentage of seed dry weight. (b) Total HFA content per seed. Data represent average and *SEM* of 10–17 biological replicates for each measurement. If letters above two bars are different, then there is significant difference between the two bars. Upper‐case letters compare the same line between light treatments; lower‐case letters compare each line within a light treatment

In addition to differences between oil amounts between 16‐hr and 24‐hr light, there were also significant differences in the amounts of the novel HFA in the transgenic lines between light treatments. Figure 2 demonstrates the seed fatty acid composition (weight percent of total fatty acids). For all transgenic lines, there was a higher percentage of HFA in the seed oil when grown under 16/8‐hr day/night cycles than for the 24‐hr continuous light. The lower percent HFA at 24‐hr light seeds was compensated for predominantly by higher levels of 18:1. While just percent fatty acid composition is a common way to report transgenic changes in seed fatty acid content, it is a measurement that is independent of the total amount of fatty acids in the seeds. Considering that light effects the quantity of seed oil, we determined the total amount of HFA as both a percentage of seed weight (Figure 3a) and as μg HFA per seed (Figure 3b). The apparent reduction in percent HFA composition when grown at 24‐hr light in Figure 2 was not actually a reduction in total HFA amount. The total amount of HFA in all transgenic lines increased in 24‐hr light conditions as compared to the day/night cycle (with the acceptation of the DGAT line measured only as a percentage of seed weight, Figure 3a). Therefore, considering that 24‐hr light increases the total amount of all fatty acids including HFA in seeds, the reduction in the percent HFA composition (Figure 2) was due to a larger increase in accumulation of common endogenous fatty acids over that of the novel HFA within the transgenic lines.

In summary, 24‐hr light produces more total fatty acids than a day–night cycle, but novel fatty acid production does not keep up with the increase in production of endogenous fatty acids, which effects the fatty acid percent composition. In addition, the reduced oil phenotypes of various transgenic lines are artificially minimized when grown under 24 hr and measured solely as oil content by weight.

### 3.2 Effect of light intensity on novel fatty acid engineering of seed oil

**Figure 4:**
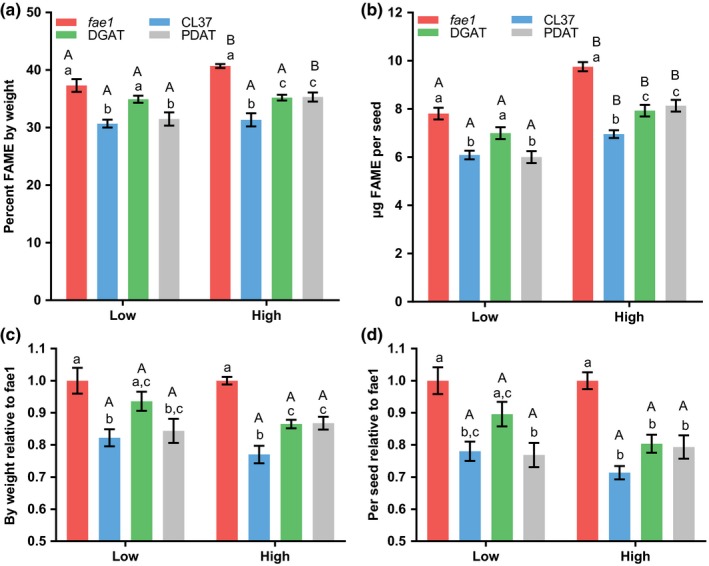
Seed oil content for day/night cycle grown at low and high light intensities. The 16/8‐hr day/night cycle light intensities in μmol photons m^−2^ s^−1^ are as follows: Low, 112; and high, 364. (a) Total seed lipid content as a percentage of seed dry weight. (b) Total lipid content per seed. Data represent average and *SEM* of 10–17 biological replicates for each measurement. (c) and (d) represent the relative lipid content of the transgenic with respect to *fae1* in a and b, respectively. If letters above two bars are different, then there is significant difference between the two bars. Upper‐case letters compare the same line between light treatments; lower‐case letters compare each line within a light treatment

To determine the effect of changing light intensity on novel fatty acid engineering, plants were grown in equivalent Percival E41HO chambers under a 16/8‐hr day/night cycle at low and high light intensities (112 and 364** **μmol photons m^−2^ s^−1^ white light, respectively) as compared to the intermediate light intensity (200 μmol photons m^−2^ s^−1^) in the day/night versus continuous light experiment (Figure 1, 2, 3). All plant lines were grown together randomized across each growth chamber. The plants of the high light treatment grew and matured 2–3 weeks faster than the low light treatment. The seed oil quantity from each plant line was measured as a percentage of seed weight (Figure 4a) and as μg per seed (Figure 4b). The differences between low and high light intensity within a 16/8‐hr day/night cycle were less clear‐cut than the day/night versus continuous light experiments and were dependent on individual line, type of oil content measurement (Figure 4a vs. 4b), and comparison method utilized Figure 4a,b vs. 4c,d).

As expected from previous light intensity experiments with Col‐0 (Li et al., 2006), the *fae1* line produced significantly more seed oil by weight in the high light treatment as compared to the low light treatment (Figure 4a). The same was also observed for the PDAT line; however, there was no statistical difference between the low and high light treatments for both the CL37 and DGAT lines when measured as a percentage of seed weight. When oil content was measured as μg per seed (Figure 4b), the high light treatment produced significantly more oil than the low light treatment for all four plant lines.

Comparing the amount of oil between the lines within a light treatment led to similar conclusions for both the oil measurement types (Figure 4a,b). At low light, the PDAT line significantly recovered the reduced oil phenotype of CL37 such that it was not statistically different than *fae1*. However, the DGAT line under low light did not recover the reduced oil phenotype of CL37 and was still significantly less than *fae1*. Under high light conditions, the amount of oil in both the PDAT and DGAT lines was significantly higher than CL37, but both were still significantly less than *fae1*. Interestingly, when the amount of oil in each transgenic line was analyzed relative to the *fae1* control within a light treatment and the subsequent transgenic/*fae1* oil content ratios compared between light treatments (Figure 4c,d), there was no statistical difference for the effect of light intensity on the relative amount of oil between the transgenics and the *fae1* control. The difference in significance of oil content between individual lines within a light treatment when comparing the analysis in Figure 4a versus Figure 4c and Figure 4b versus Figure 4d was due to the propagation of error during the ratio calculation which raised the ANOVA *p*‐values out of the significant range (e.g. <0.05).

**Figure 5:**
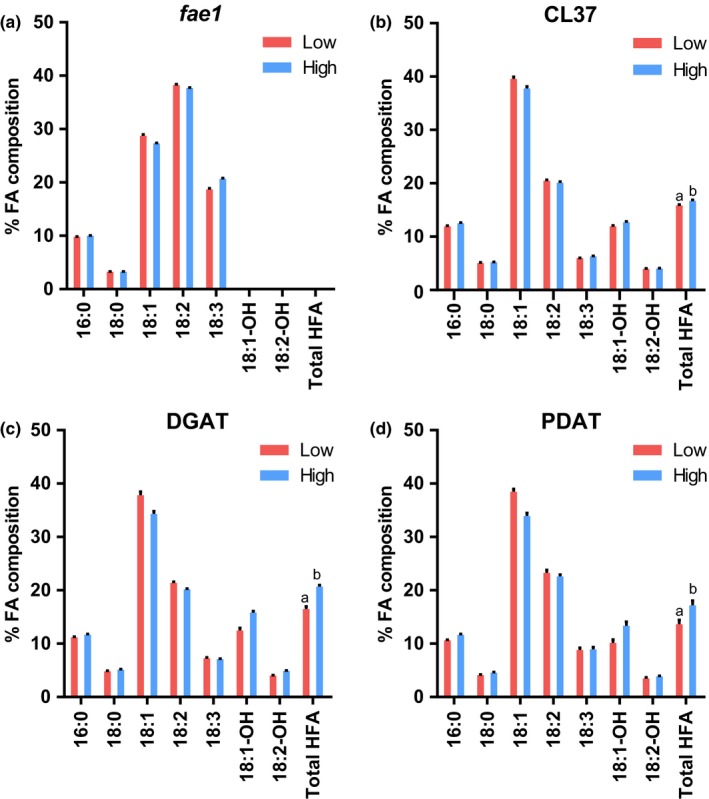
Seed fatty acid composition for day/night cycle grown at low and high light intensities. Fatty acid composition of mature seeds is percent by weight. (a) *fae1*. (b) CL37. (c) DGAT line. (d) PDAT line. Data represent average and *SEM* of 10–17 biological replicates for each measurement. The significance for total HFA content is represented by different letters above each bar. Fatty acid nomenclature: # carbons:# double bonds, ‐OH indicates presence of a hydroxyl

**Figure 6:**
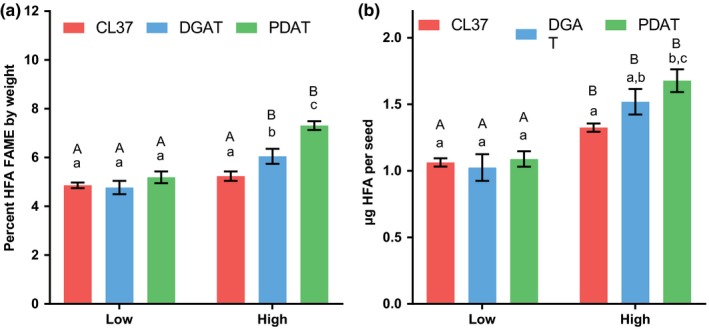
Total seed HFA accumulation for day/night cycle grown at low and high light intensities. The 16/8‐hr day/night cycle light intensities in μmol photons m^−2^ s^−1^ are as follows: Low, 112; and high, 364. (a) Total HFA content as a percentage of seed dry weight. (b) Total HFA content per seed. Data represent average and *SEM* of 10–17 biological replicates for each measurement. If letters above two bars are different, then there is significant difference between the two bars. Upper‐case letters compare the same line between light treatments; lower‐case letters compare each line within a light treatment

The effect of increasing light intensity within a 16/8‐hr day/night cycle on fatty acid percent composition of the transgenic lines had a consistent trend with the least HFA in the low light treatment and the most HFA in the high light treatment in each of the transgenic lines (Figure 5). When total HFA abundance was considered as a percentage of seed weight, there was no significant difference between the light treatments for CL37, but there was a significant increase for both the PDAT and DGAT lines (Figure 6a). When HFA accumulation was measured as μg HFA per seed, all three transgenic lines showed a significant increase in HFA accumulation at high light over the low light treatment (Figure 6b).

In summary, the high light intensity within a 16/8‐hr day/night cycle increased total fatty acid content as μg/seed in all lines as compared to low light, but the results were line specific when fatty acid accumulation was measured as a percentage of seed weight (Figure 4). Unlike the apparent decrease in percent HFA composition of seed oil in transgenic plants when the amount of light was increased by growing under 24‐hr continuous light (Figure 2), the increase in light intensity from low to high within the day/night cycle led to an increase in both the percent HFA composition (Figure 5) and in the total accumulation of HFA (Figure 6).

## 4 DISCUSSION

It has been well characterized that wild‐type Arabidopsis seed oil content is strongly dependent on light conditions (Li et al., 2006). Our results comparing a 16/8‐hr day/night cycle versus continuous light and different light intensities within a day/night cycle confirm these previous results with our nontransgenic control *fae1*. In addition, we provide additional results which indicate that various transgenic lines affected in fatty acid and oil biosynthesis do not respond to fluctuating light conditions the same as the nontransgenic control does. We also demonstrate the conclusions drawn about relative oil content between transgenic lines and the nontransgenic control, and between the different transgenic lines, are greatly dependent on the type of oil content measurement and analysis method used.

The transgenic lines utilized in these experiments have been engineered to accumulate novel HFA in Arabidopsis seed oil. However, the production of HFA has been previously characterized to induce inhibition of acetyl‐CoA carboxylase activity (ACCase, the initial step of fatty acid synthesis) and thus reduce total oil accumulation in CL37 (Bates et al., 2014). The DGAT and PDAT lines coexpressing the castor hydroxylase *RcFAH12* with either *RcDGAT2* or *RcPDAT1a* more effectively incorporate HFA into TAG have increased ACCase activity and at least partially recover the reduced oil phenotype (Bates et al., 2014). The differential accumulation of seed oil between the control and each transgenic line under various light treatments (Figures 1 and 4) is likely due to the differential strength of the HFA‐induced inhibition of ACCase between each transgenic line. The relative strength of ACCase inhibition likely limits the ability each transgenic line to upregulate of fatty acid synthesis in response to more light, as compared to the uninhibited nontransgenic control. For example, 24‐hr light or the high light day/night cycle treatment both measured as μg fatty acids per seed (Figures 1b,c and 4b,d) indicated the biggest differences in relative oil content between *fae1* and the DGAT and PDAT lines, even though the total oil content of those lines was higher than all other light treatments (Figures 1b and 4b). The likely reason for the low oil content of the transgenics relative to the nontransgenic control was that similar to wild type (Li et al., 2006) the *fae1* line has the ability to greatly increase fatty acid synthesis in response to additional light, but in each transgenic line the novel fatty acid‐induced inhibition of fatty acids (Bates et al., 2014) limits this response to varying degrees (Figures 1b and 4b). Considering that reduced seed oil content has been indicated in many different instances of novel fatty acid composition engineering (Knutzon et al., 1999; Larson et al., 2002; Li et al., 2012; Lunn et al., 2018; Mansour et al., 2014; Shrestha et al., 2016), it is likely that other novel fatty acid engineering strategies will demonstrate similar seed oil results based on light conditions.

When interpreting conclusions on the effect of novel fatty acid engineering on total seed oil accumulation, we found that both the light conditions and the type of measurement utilized can influence the conclusions drawn. The maximal amount of seed fatty acids for all lines by both measurement types was with the 24‐hr light treatment at 200 μmol photons m^−2^ s^−1^, but the differences between the lines were dependent on measurement. Seed fatty acid content as a percentage of seed weight minimized the differences between the lines such that the there was no statistical difference between the *fae1* control and both the PDAT and DGAT lines under 24‐hr light (Figure 1a). However, when fatty acid content was measured as μg per seed, each line was statistically different from each other (Figure 1b).

For plants grown under a day–night cycle, there was a general trend of a higher average oil content with higher light intensities, but the significance of the differences between light treatments was also dependent on the measurement type and the individual lines. When total fatty acids were measured as percent of seed weight, only the *fae1* and PDAT line had significant increases in the high light treatment over the low light treatment (Figure 4a), with PDAT having the largest increase in oil content from 31.5% ± 1.2 to 35.3% ± 0.8. However, the PDAT line oil content was still less than *fae1* in the same experiment which increased from 37.3% ± 1.1 to 40.7% ± 0.4. This suggests that the HFA‐induced inhibition of fatty acid synthesis is least in the PDAT line over the other two transgenic lines, but fatty acid synthesis is still inhibited as compared to the nontransgenic control. When total fatty acids were measured as μg per seed, all lines grown under high light had a significant increase in oil content over the low light‐treated plants (Figure 4b). Together these results suggest that light also affects seed weight by components other than lipids (e.g., protein, starch, and fiber), and that when focusing on lipid content alone measurements based off seed weight may not be as informative to the actual changes in lipid content as measurements on a per seed basis.

When considering the effects of just low light on the differences in oil content between the lines, low light minimized the difference between the nontransgenic control and the transgenic lines. For example, the PDAT line was not statistically different from *fae1* by either oil content measurements under low light (Figure 4a,b), but PDAT oil content was statistically less than *fae1* when measured as a percent of seed weight when plants were grown at a day/night cycle at 200 μmol photons m^−2^ s^−1^ (Figure 1a,c), and statistically less for both oil measurements for the high light treatment (Figure 4). These results suggest that suboptimal growth conditions (such as a growth chamber with old dim lights) may produce misleading results as to the ability of different plant lines to accumulate seed oils.

When seed oil content of the transgenic lines was analyzed as a ratio of the nontransgenic control, the differences between the light treatments and within each treatment by both oil measurements became less significant (Figure 1c,d and 4c,d). Only the day/night cycle versus 24‐hr light experiment when measured as fatty acid content as a percentage of seed weight was significantly different between the two treatments for all transgenic lines when analyzed as a ratio of the control (Figure 1c). All other comparisons of light treatments as a ratio of transgenics to control were not significant (Figure 1d and 2c,d). A simple conclusion would be that even though the total oil amount is changing based on light, the relative amounts of oil between lines do not change. However, this does not fit with the direct oil abundance measurements. For example, it is clear that the pattern of oil accumulation among the four lines (and the significant differences between each line) is different between the light treatments in each experiment (Figure 1a,b and 4a,b). However, the differences between the lines were less significant after the ratio calculation because the error is propagated through the division calculation. Thus, this result implies larger numbers of replicates are likely needed for seed oil content measurements when the analysis method involves mathematical manipulation of the data and propagation of the error.

An interesting result from these experiments was the difference that extra light had on accumulation of HFA between the day/night versus continuous light experiment (Figures 2 and 3) and the light intensity experiment (Figures 5 and 6). Extra light appeared to reduce the HFA content as a percentage of total fatty acids when it was provided as 24‐hr light (Figure 2), whereas extra light provided as a higher light intensity during a day/night cycle increased the percent HFA composition (Figure 5). However, both treatments increased the absolute levels of HFA in seeds for each line (Figures 3 and 6), with the exception of CL37 HFA measured just as a percent of seed weight for the low versus high light treatment (Figure 6a). The effect of light conditions on percent fatty acid composition (Figures 2 and 5) is likely due to the differences in rate of fatty acid synthesis versus fatty acid modification. Previously it was demonstrated that high rates of fatty acid synthesis proceed predominantly in the light (Browse et al., 1981), while the slower fatty acid desaturation can also continue in the dark leading to enhanced levels of desaturated fatty acids at the end of the night period versus at the end of the photoperiod (Maatta et al., 2012). Fatty acid hydroxylation is a variant of fatty acid desaturation (Vandeloo et al., 1995) and is likely slow relative to fatty acid synthesis as well. Therefore, in the continuous light plants, fatty acid hydroxylation likely cannot keep up with fatty acid synthesis to generate the same proportion of HFA as the during the day/night cycle. Likewise, the higher percent HFA with increasing light intensity with 16/8 day/night cycles is likely due to the enhanced production of the 18:1 substrate during the day, and during the subsequent night period the hydroxylase catches up and produce more HFA. When the HFA content between the three transgenic lines was compared just within the low light treatment, there were no statistical differences in HFA content between the CL37, DGAT, and PDAT lines. Therefore, suboptimal light conditions can minimize the effect of gene stacking engineering strategies to increase total levels of the desired product.

In conclusion, we demonstrate that the relative “success” of fatty acid engineering strategies for amount of novel fatty acids, their proportion within the oil, and their effect on total oil content relative to the oil content of controls and other engineered lines is highly dependent on light conditions, the type of oil content measurement, and data analysis methods (e.g., as exact values or as a ratio relative to the control). Therefore, our recommendations to the Arabidopsis seed oil engineering community are as follows: 1) to consider measuring seed oil and novel fatty acid quantity as part of the analysis of fatty acid engineering experiments rather than just fatty acid percent composition. 2) To utilize a day/night cycle growth condition instead of continuous light for studies quantifying seed oil content. Even though continuous light helps the plants to grow faster, it can lead to misleading results on both fatty acid composition and oil content as compared to nontransgenic controls and other transgenic lines. 3) To measure both oil content as a percentage of seed weight and oil content per seed because each has value and can provide alternative conclusions about the changes in oil content due to engineering. Finally, 4) to include detailed reporting of Arabidopsis growth conditions for each experiment in publications for the critical analysis of the success of oilseed engineering strategies between publications within the literature.

## AUTHOR CONTRIBUTIONS

Both NK and PB planned experiments. NK conducted experiments. Both NK and PB analyzed data and wrote the manuscript.
