# Supplementary material for: The effect of light conditions on interpreting oil composition engineering in Arabidopsis seeds
Source: Plant Direct. 2018 Jun 26;2(6):e00067. doi: 10.1002/pld3.67 (PMC6508571; doi:10.1002/pld3.67)
Supplement: Supplementary file 1 [file PLD3-2-e00067-s001.pdf]

Response to the Reviewer's response

Reviewer #1 :

This manuscript by Karki and Bates addresses an important aspect of seed lipid metabolism-the effects of the light environment on seed oil accumulation. It is well known, but perhaps not widely appreciated that light intensity and duration can substantially affect the oil content in seeds. Here the authors demonstrate this and investigate this phenomenon in the context of hydroxy fatty acid accumulation using transgenic Arabidopsis lines previously developed. The results are consistent with previous work and follow expectations for the most part. Some useful insights are pointed out here and the authors make some suggestions about using and reporting the various light conditions for oilseed studies. I have a couple of concerns that the authors may wish to address.

1. The "control" used here is a fae1 knockout, which already has an altered fatty acid composition compared to "wild-type" Arabidopsis. Shouldn't a wild-type Arabidopsis genotype be used here as a reference?

The goals of this experiment were to determine the effect of transgenic production of novel fatty acids in Arabidopsis seeds on the ability of the plant to alter oil accumulation in response to light. The genetic background for the 3 transgenic lines used in this study is the *fae1* mutant, therefore it is the proper non-transgenic control. Therefore, differences between *fae1* and the transgenic lines will be due to the transgenes and not the mutant itself. In addition, wild-type has been previously well characterized to adjust seed oil abundance based on light, and previous measurements of *fae1* vs wild-type oil content has demonstrated them to be very similar. These references are included in the manuscript, and an enhanced description of the lines used in this study, why they were chosen, and their background is in lines 130-147. For clarity to the reader we have changed references to *fae1* from "wild-type control" to the "non-transgenic control" throughout the manuscript. As suggested by Reviewer 2 "Table 1" was included that summarizes the genetic background of the lines used and their references.

2. It seems that the three light intensity treatments (1L, 2L, and 3L) may have been influenced by the different type of chamber used. That is the 2L had lower amount of oil than either 1L- or 3L- treated plants (Fig 3A). The authors offer a reasonable explanation, such as a potential factor of humidity. But should this be reconciled here? Otherwise we run the risk of making growth recommendations before optimal growth parameters are clearly established?

We agree with the reviewers that different chambers likely have effects other than just light, therefore we have restricted our comparisons to just plants grown in the same type of chamber with only light differing. We have taken the suggestion of Reviewer 2 and removed the 2L dataset because it was done in a different type of chamber. For flow of the manuscript we have renamed 1L as "Low light" and 3L as "High light".

3. Somewhat related to number one, are these results unique to hydroxy fatty acid accumulation or to fae1 loss-of-function? Would other conclusions be reached in other types of fatty acid modifications or in other mutant backgrounds (like fad2 for example)?

As indicated in response to question 1, the comparison of the 3 transgenic lines in the *fae1* background to the *fae1* non-transgenic control indicates that the differential responses between the four lines are due to the transgenes which are involved in the production and accumulation of novel fatty acids in seed oil. The focus of this manuscript is on the effects of novel fatty acid engineering, and we cite several other papers which have characterized a change in seed oil content from the engineering (Lines 84-87, 269-273) therefore it is likely that similar differential responses to light between transgenics and their control backgrounds will exist for other novel fatty acid engineering. It is also likely that some fatty acid composition changes will lead to no adverse changes in oil content. This is one of the main points of the manuscript, to point out to the plant lipid community that careful analysis of oil amount (not just percent fatty acid composition) is important when determining the effects of engineering a change in seed fatty acid composition. This point was added to the recommendations to the community in the conclusion (lines 344-346). There are many possible changes in seed oil fatty acid composition that could be analyzed in this manner and it is beyond the scope of this manuscript to investigate them all, we hope that this manuscript will be helpful for the plant lipid community to design future experiments and to critically evaluate those in the literature.

Minor comments:
Line 140- umole change to umol

Changed

Line 174- "when under 24 hr and measured solely"; change to "when grown 24 hr light and measured solely"

Changed

Line 202- insert in brackets for clarity, "(Fig. 3C,D), [showed] the best recovery at 2L, and [had] a minimal".

Sentence removed along with the data as suggested by Reviewer 2.

Line 206- Change "Despite the growth line specific differences that increasing" to "Despite the line-specific growth differences that increasing".

Sentence rewritten, now lines 226-228.

Lines 209-211- Correct sentence for clarity and grammar to .... "In summary, compared to the fae1 wild-type control, light intensity at the lower end of the optimal growth range for Arabidopsis (1L, 112 µmol photons m-2 s-1) minimized the negative affect that HFA production had on oil content within CL37, and this was further enhanced by PDAT, but not by DGAT."

That paragraph was rewritten now as lines 233-239.

Lines 212-214, please clarify

That paragraph was rewritten now as lines 233-239.

Line 340- the data [are] presented.

Changed

The discussion as a whole could use another round of editing for clarity and grammar.

The discussion has been mostly rewritten to account for the changes in data and analysis suggested by Reviewer 2.

Reviewer #2 :

According to previous studies, Arabidopsis CL37 plant overexpressing RcFAH12 in a background of fae1 mutant accumulates hydroxyl fatty acid (HFA) but reduces oil content in seeds. In this study, Karki and Bates investigated the photoperiod and the light intensity for plant growth to improve seed oil content and/or HFA composition. Results demonstrate that the continuous light produced more oil content but induced a lower percentage of HFA compared with the long day photoperiod. The low light intensity waved the reduction of lipid content in CL37 and the high light intensity enhances the increase of HFA composition. Overall, this study provided us the importance of deciding the plant growth conditions that affect on the oil content and unusual fatty acid composition in Arabidopsis transgenic lines producing HFA. I have some suggestions and comments that can improve the manuscript.

Major comments
1, Page 5, lines132-134:
Here, authors suddenly decided to use the fae1 mutant that defects in the synthesis of very long chain fatty acids as 'non-transgenic wild-type control' without any explanation of this background. For readers who are not familiar in this field, authors should justify the use of fae1 mutant here or in the introduction part. Also, fae1 mutant lacking the fatty acid elongase should not be referred to as the wild type in the text. It would be helpful if authors could prepare a figure or a table explaining genotypes of all lines used in this study.

The goals of this experiment were to determine the effect of transgenic production of novel fatty acids in Arabidopsis seeds on the ability of the plant to alter oil accumulation in response to light. The genetic background for the 3 transgenic lines used in this study is the *fae1* mutant, therefore it is the proper non-transgenic control. Therefore, differences between *fae1* and the transgenic lines will be due to the transgenes and not the mutant itself. In addition, wild-type has been previously well characterized to adjust seed oil abundance based on light, and previous measurements of *fae1* vs wild-type oil content has demonstrated them to be very similar. These references are included in the manuscript, and an enhanced description of the lines used in this study, why they were chosen, and their background is in lines 130-147. For clarity to the reader we have changed references to *fae1* from the "wild-type control" to the "non-transgenic control" throughout the manuscript. As suggested by Reviewer 2 "Table 1" was included that summarizes the genetic background of the lines used and their references.

2, Page 6, lines 172-175:
Authors concluded that HFA accounts for the lower percent upon continuous light. I wonder if HFA percent per seed weight and/or amount (µg) per seed in 16 hours light are still higher than those in 24

hours light or not. This measurement might serve authors aim of this study rather than judge only with the mole percent of HFA.

We thank the reviewer for this suggestion and therefore made a new figure (Figure 3), which demonstrates the total amount of HFA as per seed weight and µg per seed. As indicated in the figure, the total amount of HFA actually increases at 24 hr light (along with total oil), but the % of HFA composition in Figure 2 decreases because other FA in the oil increase more than HFA. This is further discussed in the discussion section. In addition we did the same analysis for the various light intensity experiment which is the new Figure 6.

3, Figure 1 and 3:
Although the result of 16 hours light in Figure 1 (200 µmol photons m-2 s-1, AR22L) and the result of 2L in Figure 3 (218 µmol photons m-2 s-1, AR75L2) were obtained upon similar condition, there are the unignorable differences in results between Figure 1 and 3. Especially, the percentages of seed weight of four Arabidopsis lines in Figure 3A 2L are much lower than those in Figure 1A 16 hours (around 5-10%). It seems like that seed weights increased in the plants grown under 2L in AR75L2, and thus, these two experiments are lack of consistency.
Furthermore, authors used Percival E41HO for 1L and 3L, and Percival AR75L2 for 2L to perform the light intensity experiment, and concluded that 'the least amount of oil for the fae1, CL37 and DGAT lines was in the 2L treatment (Page 6, lines 192-193)'. Here, as authors mentioned in the discussion part, I suspect that there might be some environment differences between these two incubators, such as light source or the aeration which affect humidity and CO2 condition. To conclude regarding with 2L, authors should repeat the result of 2L using Percival E41HO chamber. Alternatively, removing 2L from Figure 3 and mentioning the result of Figure 1 as the intermediate light intensity might be another solution.

We agree with the reviewers that different chambers likely have effects other than just light, therefore we have restricted our comparisons to just plants grown in the same type of chamber with only light differing. We have taken the suggestion of Reviewer 2 and removed the 2L dataset because it was done in a different type of chamber. For flow of the manuscript we have renamed 1L as "Low light" and 3L as "High light".

Minor comments
Authors mixed uppercase letters and lowercase letters as referring to each figure content in the text. Please choose one of them in the whole manuscript (Fig. 1a or Fig. 1A).

This has been changed.

Page 5, lines 149-150:
'In general, the continuous light treatment produced more oil by both measurements.'
The reference is missing, please include it.

The reference to Fig. 1a,b was included.

Figure 1 and 3:
There is no statistical analysis of data shown in Figure 1C-D, Figure 3C-D. Please include it.
Note: Previous Figure 3 is now Figure 4.
For both figures for simplicity the "Relative to fae1 data" was changed from a percent value to a ratio

with fae1 set at "1". This data is essentially the oil content measurements for each line divided by that for fae1 within a light treatment experiment. The propagation of error through ratio calculations $A\Delta a / B\Delta b = C\Delta c$ was done by the two formulas, where the ratio is $C = A/B$, and the standard deviation is $\Delta c = C\times\sqrt{((a\div A)^2+(b\div B)^2)}$. This calculation was added into the methods (lines 392-394) and statistical analysis (Two-way ANOVA) was completed for this dataset as for the other data sets. These new results lead to additional discussion on the changes in significance when error is propagated through calculations in the results (lines 223-225) and in the discussion (304-317).
